# Functional Characterization of the γ-Aminobutyric Acid Transporter from Mycobacterium smegmatis MC^2^ 155 Reveals Sodium-Driven GABA Transport

**DOI:** 10.1128/JB.00642-20

**Published:** 2021-01-25

**Authors:** Ana Pavić, Yurui Ji, Agnese Serafini, Acely Garza-Garcia, Martin J. McPhillie, Alexandra O. M. Holmes, Luiz Pedro Sório de Carvalho, Yingying Wang, Mark Bartlam, Adrian Goldman, Vincent L. G. Postis

**Affiliations:** a Astbury Centre for Structural Molecular Biology, School of Biomedical Sciences, University of Leeds, Leeds, United Kingdom; b Biomedicine Research Group, Faculty of Health and Social Sciences, Leeds Beckett University, Leeds, United Kingdom; c College of Environmental Sciences & Engineering, Nankai University, Tianjin, China; d Mycobacterial Metabolism and Antibiotic Research Laboratory, The Francis Crick Institute, London, United Kingdom; e School of Chemistry, Faculty of Engineering & Physical Sciences, University of Leeds, Leeds, United Kingdom; f College of Life Sciences, Nankai University, Tianjin, China; g Molecular and Integrative Biosciences, University of Helsinki, Helsinki, Finland; Rutgers University-Robert Wood Johnson Medical School

**Keywords:** GABA, membrane biology, mycobacteria, transporter

## Abstract

Characterizing the mycobacterial transporters involved in the uptake and/or catabolism of host-derived nutrients required by mycobacteria may identify novel drug targets against tuberculosis. Here, we identify and characterize a member of the amino acid-polyamine-organocation superfamily, a potential γ-aminobutyric acid (GABA) transport protein, GabP, from Mycobacterium smegmatis. The protein was expressed to a level allowing its purification to homogeneity, and size exclusion chromatography coupled with multiangle laser light scattering (SEC-MALLS) analysis of the purified protein showed that it was dimeric. We showed that GabP transported γ-aminobutyric acid both *in vitro* and when overexpressed in E. coli. Additionally, transport was greatly reduced in the presence of β-alanine, suggesting it could be either a substrate or inhibitor of GabP. Using GabP reconstituted into proteoliposomes, we demonstrated that γ-aminobutyric acid uptake is driven by the sodium gradient and is stimulated by membrane potential. Molecular docking showed that γ-aminobutyric acid binds MsGabP, another Mycobacterium smegmatis putative GabP, and the Mycobacterium tuberculosis homologue in the same manner. This study represents the first expression, purification, and characterization of an active γ-aminobutyric acid transport protein from mycobacteria.

**IMPORTANCE** The spread of multidrug-resistant tuberculosis increases its global health impact in humans. As there is transmission both to and from animals, the spread of the disease also increases its effects in a broad range of animal species. Identifying new mycobacterial transporters will enhance our understanding of mycobacterial physiology and, furthermore, provides new drug targets. Our target protein is the gene product of *msmeg_6196*, annotated as GABA permease, from Mycobacterium smegmatis strain MC^2^ 155. Our current study demonstrates it is a sodium-dependent GABA transporter that may also transport β-alanine. As GABA may well be an essential nutrient for mycobacterial metabolism inside the host, this could be an attractive target for the development of new drugs against tuberculosis.

## Abstract (precis)

The spread of multidrug-resistant tuberculosis increases its global health impact in humans. As there is transmission both to and from animals, the spread of the disease also increases its effects in a broad range of animal species.

## INTRODUCTION

Tuberculosis (TB), one of the oldest and deadliest human diseases, is caused by Mycobacterium tuberculosis. M. tuberculosis is a leading infectious killer, claiming the lives of about 1.2 million people annually (1). It is a reemerging pathogen, due to the development of multiple-drug resistant (MDR) and extensively drug resistant (XDR) strains (2). In addition, animal tuberculosis is a globally distributed zoonotic chronic disease, posing a significant impact in global agricultural losses (3). Since transmission between humans and animals has been demonstrated in both directions, TB has been described as a One Health issue, having similar consequences for humans and animals (4), and causing huge socioeconomic impact both in terms of human lives and resources.

The unique cell wall composition of M. tuberculosis is believed to be the major determinant of its resistance to a large range of antibiotics (5), leading to a continuing need to discover new ones. A new approach to developing therapeutics against TB is through comprehensive discovery and characterization of metabolic pathways, their impact on key features of M. tuberculosis pathogenesis (6), and their contribution to drug resistance. Because M. tuberculosis has a reduced genome (7), it has neither extended *de novo* synthesis pathways nor duplicated transporters, i.e., the essential transmembrane proteins that mediate nutrient uptake and metabolite efflux (8), meaning there are many essential metabolites. The transporters, required for the uptake of essential nutrients, enable mycobacteria to persist in the harsh intracellular host environments by scavenging nutrients. They thus represent potential new drug targets. Characterizing unique mycobacterial transporters provides another route to discover novel therapeutics to treat TB (9).

Despite considerable progress in the development of genetic methods for mycobacteria, the function of many transporters is still unknown. Nonetheless, AnsP1, an aspartate importer, was identified by targeted mass-spectrometry-based metabolomics. This method targets a predefined group of compounds and aims to determine which one is transported. A mixture of biomolecules is introduced into the mass spectrometer either directly or following a separation procedure. In particular, liquid chromatography–mass spectrometry-based targeted metabolomics achieves high-level sensitivity and accuracy (10). AnsP1 was shown to be involved in nitrogen metabolism and essential for mycobacterial infection and survival (11–13). This provides evidence that M. tuberculosis relies on amino acid uptake and degradation pathways to thrive inside the host and confirms a strong link between nutrition and pathogenicity in M. tuberculosis.

To identify the substrates of other orphan transporters from mycobacteria, we adopted a similar approach for five homologous genes of AnsP1 from three different mycobacterial species. From this initial screen, the annotated γ-aminobutyric acid (GABA) permease from Mycobacterium smegmatis MC^2^ 155 (MsGabP hereafter) proved promising for further studies. M. smegmatis is widely used as a convenient model system to study M. tuberculosis biology, cell structure, and persistence under conditions of nutrient starvation (14) because it has a higher growth rate and lower biosafety level than M. tuberculosis. Based on its sequence, this putative permease belongs to the amino acid-polyamine-organocation (APC) superfamily of transport proteins (15), which is widely found in all living organisms. To date, no *in vivo* or *in vitro* information is available for Mycobacterium smegmatis GabP despite its being annotated as a probable GABA permease based on its sequence identity to Escherichia coli GabP (16). The hydropathic profile produced by the TMHMM server predicted that MsGabP has 12 transmembrane-spanning helices (17). Homology modeling of the protein shows that it adopts an arrangement known as the LeuT fold (18) (see Fig. S1 in the supplemental material).

In eukaryotes, besides its primary function as an inhibitory neurotransmitter (19), GABA may have a role in the modulation of immune responses (20) but it is unclear whether and how GABAergic signaling regulates antimicrobial host defenses during infections. GABA does, however, act as a specific cytotoxicity and virulence regulator of Pseudomonas aeruginosa (21). Bacteria, such as Bacillus subtilis (22), P. syringae (23), and isolated E. coli mutants (24), can use GABA as a sole nitrogen source. Furthermore, GABA is known to be important for acid resistance in bacteria, including E. coli (25) and *Lactobacillus* (26). In the M. tuberculosis vaccine strain Mycobacterium bovis BCG, uptake of GABA was not induced by carbon and nitrogen starvation (27). Beyond this, the mechanism and implications of GABA transport and metabolism have not been extensively examined in mycobacteria.

We report here the successful expression of M. smegmatis MC^2^ 155 GabP in E. coli. SEC-MALLS analysis of the purified protein showed that it is a homodimer. Both *in vitro* and in live recombinant bacteria, functional experiments allowed us to confirm predictions that it is a GABA transporter. We also showed that substrate uptake by MsGabP is sodium driven and depends on the membrane potential.

## RESULTS

### MsGabP expressed in E. coli.

**FIG 1:**
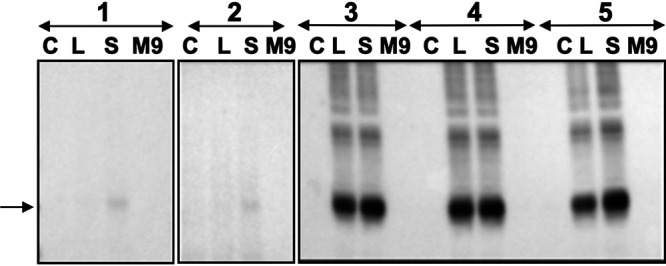
Expression of MsGabP. The expression of MsGabP in E. coli was analyzed by Western blotting (band migrating at ∼36 kDa indicated by an arrow, detected by anti-HIS antibody). Lanes represent MsGabP expression results in C41 (DE3) Δ*acrB* pRARE2 (1), C43 (DE3) Δ*acrB* pRARE2 (2), C41 (DE3) Δ*acrB* (3), BL21 Star (DE3) pRARE2 (4), and BL21 Gold (DE3) pRARE2 (5). Medium types are labeled as follows: C, LB medium containing glucose; L, LB autoinduction medium; S, SB autoinduction medium; M9, M9 autoinduction medium. The gels were spliced for labeling purposes.

Expression of MsGabP was induced in E. coli under the control of the IPTG (isopropyl-β-d-thiogalactopyranoside)-inducible promoter Tac. The expression level was tested by Western blot analysis in five strains and three different media (Fig. 1). In strains C41 (DE3) Δ*acrB* pRARE2 or C43 (DE3) Δ*acrB* pRARE2, we observed only a very low level of expression in superbroth (SB) medium (Fig. 1, lanes 1S and 2S). Conversely, in the other strains, the expression level was higher when the cells were cultured in SB (Fig. 1, lanes 3S, 4S, and 5S) than in LB medium (Fig. 1, lanes 3L, 4L, and 5L). Densitometry measurements (see Fig. S2 in the supplemental material) confirmed that MsGabP shows the highest expression with BL21 Gold (DE3) pRARE2 as the strain in SB autoinduction medium. The expression level with C41 (DE3) Δ*acrB* in SB autoinduction medium was about 65% of that level (Fig. 1, lanes 3S and 5S). We nonetheless chose this last condition for further optimization because AcrB, which is a known contaminant of purified membrane protein from E. coli, is deleted in this last strain. Following further optimization (data not shown), we determined that the optimal expression conditions in C41 (DE3) Δ*acrB* strain was SB autoinduction medium for 24 h at 37°C.

The protein eluted at approximately 36 kDa on sodium dodecyl sulfate-polyacrylamide gel electrophoresis (SDS-PAGE) despite that the predicted molecular mass is 50.7 kDa (see “Purification of MsGabP” in Materials and Methods).

### Targeted metabolomics suggest GABA and serine as potential substrates of MsGabP.

**FIG 2:**
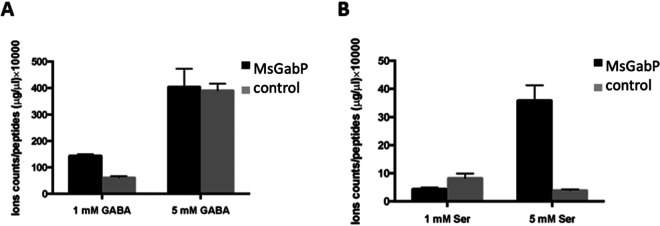
Targeted metabolomics study results of GABA (A) and serine (B). The amino acids were tested with two concentrations, 1 mM and 5 mM. Bars represent uptake resulting from cells harboring plasmid with MsGabP or the empty vector control. The graphs show means (bars) and standard deviations (error bars) from three biological replicates of one independent experiment. They are representative of two independent experiments. *y* axes report the ion counts normalized on residual protein content.

We used targeted metabolomics to identify potential substrates (see Materials and Methods). We expressed MsGabP in E. coli using the pL33 plasmid and tested for uptake of following amino acids: GABA, arginine, lysine, aspartate, asparagine, glutamine, glutamate, and serine. The rationale was as follows: (i) GABA because MsGabP is annotated as a GABA permease; (ii) arginine and lysine because MsGabP belongs to the APC superfamily of transporters; (iii) aspartate, asparagine, glutamine, and glutamate because MsGabP has about 35 to 36% identity to M. tuberculosis AnsP1 and AnsP2, which transport asparagine; and (iv) serine, which is polar but not charged, as a negative control. We observed a 2-fold differential in internal GABA concentration with 1 mM GABA in energized E. coli cells containing MsGabP (Fig. 2A). Similarly, with 5 mM serine, there was about a 7-fold differential (Fig. 2B), suggesting these two amino acids could represent potential substrates. No transport of the other tested amino acids was detected. For instance, neither 1 mM nor 5 mM arginine and/or aspartate increased the concentration of the respective amino acid in the MsGabP cells compared to control cells (Fig. S3).

### MsGabP transports radiolabeled GABA into cells.

**FIG 3:**
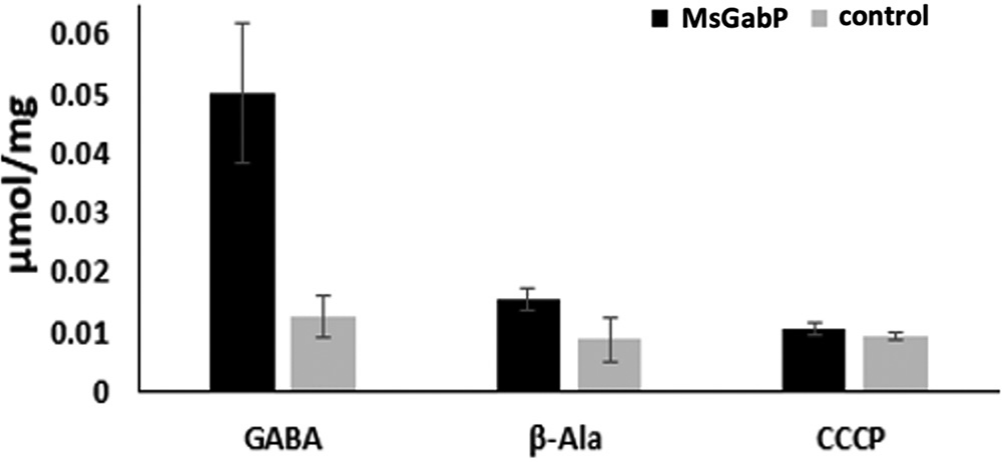
Direct measurement of [^3^H]GABA uptake into E. coli cells. The transport assay was conducted in the presence of [^3^H]GABA (GABA), unlabeled 5 mM β-alanine added before the addition of [^3^H]GABA (β-Ala), and 50 μM CCCP added before the addition of [^3^H]GABA (CCCP). Bars represent the GABA uptake from cells harboring plasmid with the GabP gene or empty vector. The data are means of three measurements. Error bars indicate standard deviations.

To confirm the uptake of potential amino acids by MsGabP, we next performed radiolabeled transport assays on intact cells. Consistent with the metabolomics study results, E. coli cells overexpressing MsGabP were able to take up radioactive GABA (Fig. 3). As a control, cells with empty plasmids showed only a negligible uptake of radioactive GABA. This clearly shows that the uptake of GABA was indeed due to the overexpressed MsGabP and not linked to E. coli endogenous GABA transporters. In contrast, radioactive serine was not transported by MsGabP, suggesting that serine is not a true substrate of the transporter (data not shown). Interestingly, β-alanine, a structural analogue of GABA, competed with the uptake of [^3^H]GABA into energized E. coli whole cells (Fig. 3), suggesting that it might bind as a competitive inhibitor of GABA. GABA uptake was also significantly decreased when a protonophore, 50 μM carbonylcyanide *m*-chlorophenylhydrazone (CCCP), was added with GABA to the assay (Fig. 3), indicating that the transport depends on the proton gradient and/or on the membrane potential.

### Purification of MsGabP.

**FIG 4:**
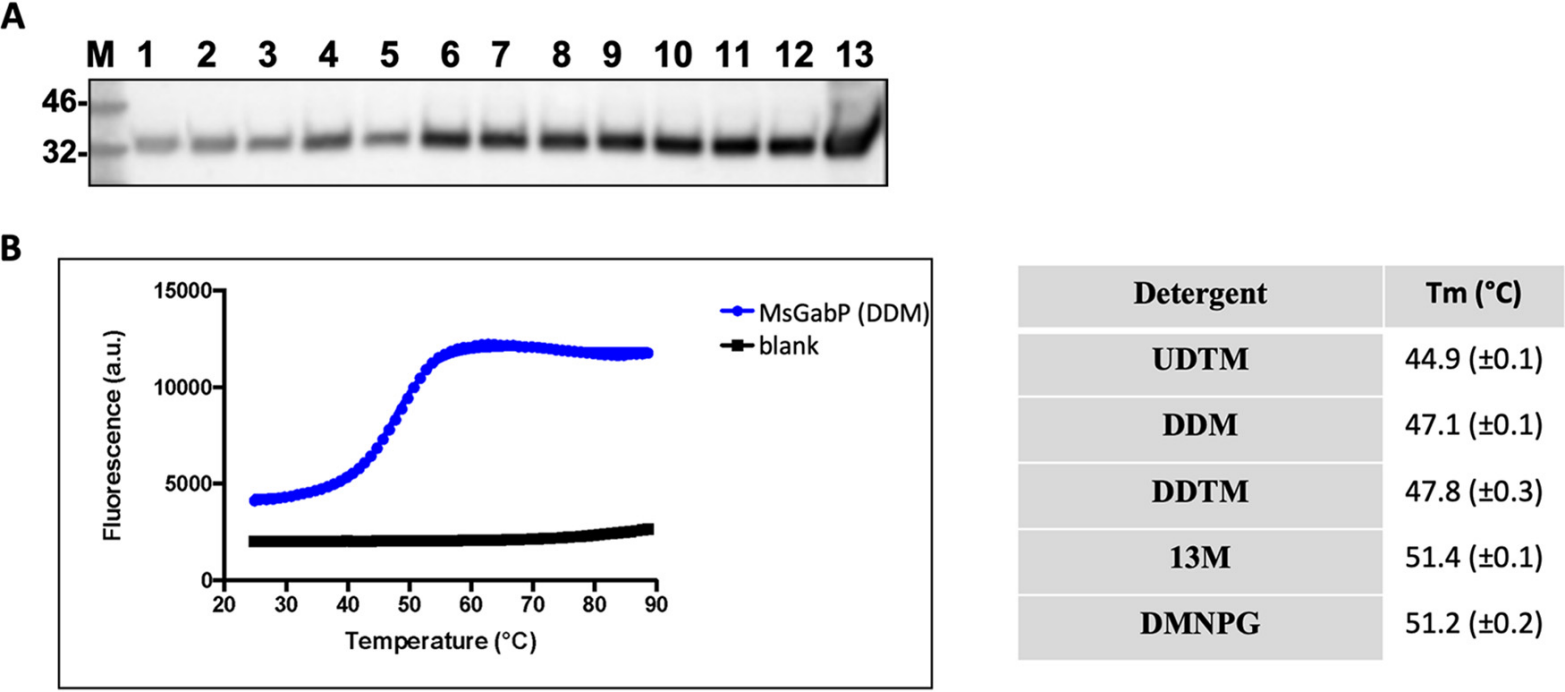
Solubilization of MsGabP. (A) Detergent solubilization screen with anti-His Western blotting of the supernatant fractions following solubilization with CYMAL 6 (lane 1), LMNPG (lane 2), DMNPG (lane 3), OGNPG (lane 4), β-OG (lane 5), 14M (lane 6), 13M (lane 7), DDTM (lane 8), DDM (lane 9), UDTM (lane 10), UDM (lane 11), and DM (lane 12). Lane 13 is a membrane fraction with no detergent added; M represents the protein molecular marker (in kilodaltons). (B) Microscale fluorescent thermal stability assay of MsGabP. GabP purified with DDM at pH 7.0. Shown are data points collected during the MsGabP unfolding process (blue) and from buffer blank control samples (black). The table shows the summary of *T_m_* values calculated from the melting curves. Values shown are the means of two separate measurements.

We next wanted to study the transport of amino acids with purified and reconstituted MsGabP and we therefore tested a range of detergents for solubilization efficiency. *n*-Decyl-β-d-maltopyranoside (DM), *n*-undecyl-β-d-maltopyranoside (UDM), *n*-undecyl-β-d-thiomaltopyranoside (UDTM), and *n*-dodecyl-β-d-maltoside (DDM) at 1% all extracted MsGabP from the membranes with similar efficiencies (∼80%), as quantified after Western blot analysis (Fig. 4A). Since DDM has a lower critical micellar concentration (CMC) than DM, we decided to proceed with DDM for solubilizing the protein in large-scale purification.

Preliminary purifications in DDM showed the purified protein was unstable and contained contaminants (not shown). We therefore used the HRV-3C-His tag to perform on-column detergent exchange followed by on-column cleavage to elute MsGabP to identify detergents that stabilized the purified protein. To identify the best one, small-scale purifications were conducted with 12 different detergents (Table 1). The stability was then assessed by microscale fluorescent thermal stability assay (28). The protein unfolded cooperatively in DDM, decyl maltose neopentyl glycol (DMNPG), UDTM, *n*-dodecyl-β-d-thiomaltopyranoside (DDTM), and *n*-tridecyl-β-d-maltopyranoside (13M) (Fig. 4B) under the buffer conditions tested. This suggests that MsGabP is correctly folded in all of these detergents. However, it was most stable in 13M or DMNPG (melting temperature [*T_m_*] of 51.4 ± 0.1°C and 51.2 ± 0.2°C, respectively) (Fig. 4B), which was about 4°C higher than in DDM (Fig. 4B). As the final yield from DMNPG was higher than from 13M (Table 1), we used DMNGP for further experiments. Yields of ∼0.7 mg protein per liter of bacterial culture were typically achieved.

**TABLE 1:** Detergents tested for solubilization efficiency and MsGabP yield after detergent exchange

Detergent^*a*^	Concn (× CMC)^*b*^	Concn (%)	MsGabP yield after detergent exchange (%)^*c*^
DDM	5.7	0.05	100
CYMAL 6	3	0.084	92.3
β-OG	3	1.59	0
OGNPG	3	0.174	35.9
DMNPG	3	0.0102	83.7
LMNPG	3	0.003	0
DM	3	0.261	71.8
UDM	5	0.145	84.8
UDTM	5	0.055	90.9
DDTM	5	0.013	51.5
13M	5	0.0085	72.7
14M	5	0.0027	0

a DDM, *n*-dodecyl-β-d-maltoside; CYMAL, 6-cyclohexyl-1-hexyl-β-d-maltoside; β-OG, *n*-octyl-β-d-glucopyranoside; OGNPG, octyl glucose neopentyl glycol; DMNPG, decyl maltose neopentyl glycol; LMNPG, lauryl maltose neopentyl glycol; DM, *n*-decyl-β-d-maltopyranoside; UDM, *n*-undecyl-β-d-maltopyranoside; UDTM, *n*-undecyl-β-d-thiomaltopyranoside; DDTM, *n*-dodecyl-β-d-thiomaltopyranoside; 13M, *n*-tridecyl-β-d-maltopyranoside; 14M, *n*-tetradecyl-β-d-maltopyranoside.

b CMC, critical micellar concentration.

c Values show protein yield after detergent exchange. The number was calculated using the amount of MsGabP obtained with 11 different detergents divided by the amount obtained from purification with DDM.

**FIG 5:**
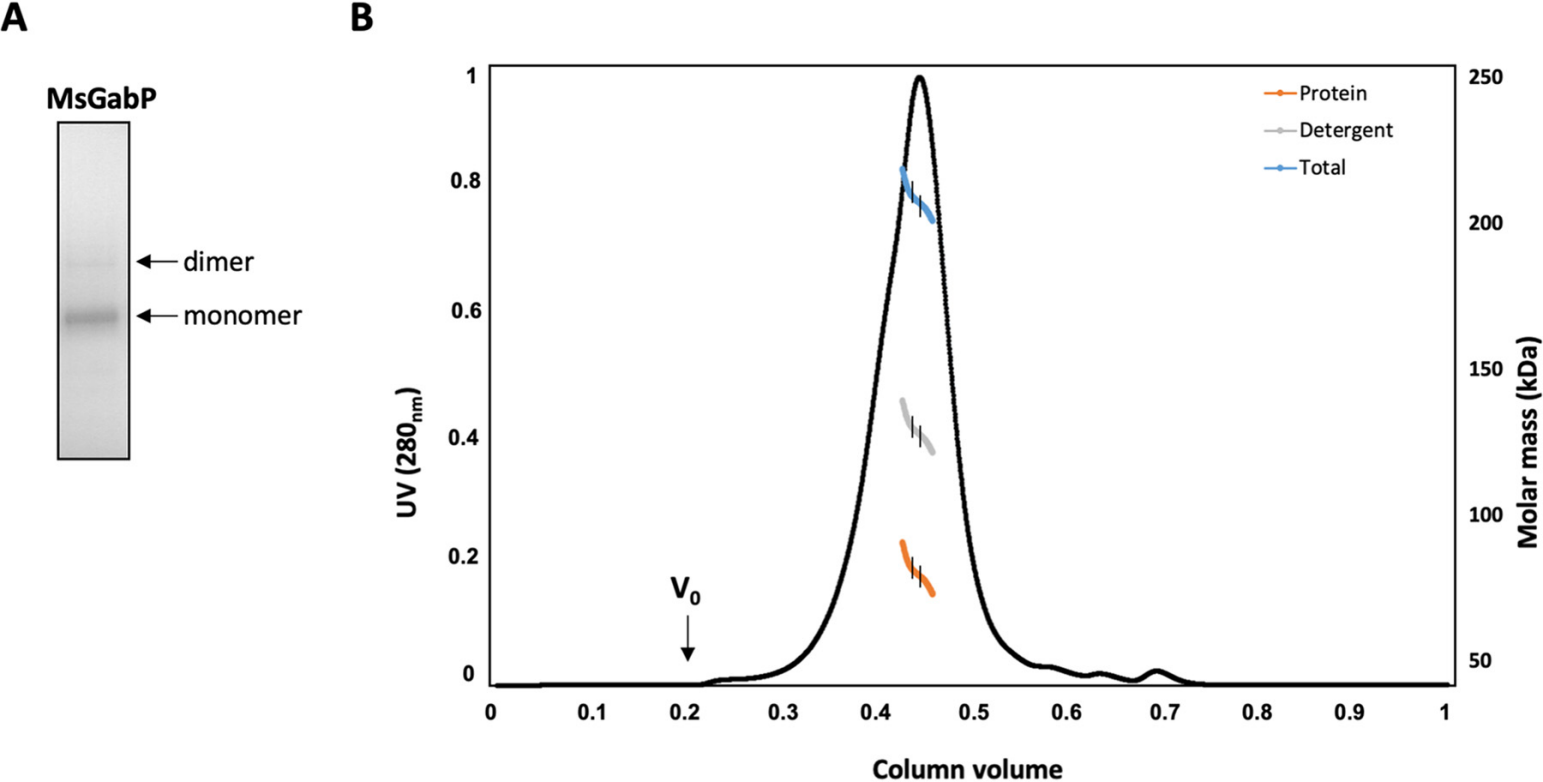
SEC-MALLS analysis of purified MsGabP. (A) SDS-PAGE of MsGabP eluted in DMNPG. The arrows indicate the bands on the gel corresponding to MsGabP at ∼36 kDa and ∼70 kDa. (B) SEC-MALLS UV chromatogram. The colored lines indicate the molar mass distribution of the eluting protein, detergent, and total complex. Vertical black lines indicate the part used for calculations of molar masses.

Fractions collected during the protein elution from the resin showed the presence of two bands, migrating at ∼36 kDa and ∼70 kDa, respectively (Fig. 5A). Peptides generated by trypsin digestion of the protein bands were analyzed using mass spectrometry. The acquired spectra were analyzed in PEAKS software, which allowed us to identify MsGabP with 12 unique peptides and a total sequence coverage of 14% in the lower band at 36 kDa and 2 unique peptides and a total sequence coverage of 3% in the upper band at 70 kDa (Fig. S4). In the lower 36-kDa band, we were able to detect peptides from the N terminus, the middle of the MsGabP, and the C terminus, consistent with a full-length protein and further supported by an active role in GABA transport (see below). The 70-kDa band is either a protein homodimer or a partially folded monomer, but most likely the latter (29).

### Purified MsGabP is a dimer.

The absolute molecular weight of the purified protein-detergent complex was determined by SEC-MALLS (Fig. 5B). The protein eluted as a single, symmetrical peak at ∼0.4 column volume, demonstrating its homogeneity and monodispersion (Fig. 5B). Using the three-detector method (30), the molecular mass of MsGabP in the main peak was found to be 79.2 kDa ± 5.7%, indicating the protein is dimeric in the protein-detergent complex.

### MsGabP-driven GABA transport depends on both Na^+^ and membrane potential.

To study MsGabP function free from E. coli metabolism and interference by its endogenous amino-acid transporters, we reconstituted purified MsGabP into liposomes. The migration (“flotation”) of proteoliposomes on a discontinuous sucrose gradient demonstrated successful reconstitution of the protein into liposomes with an efficiency of ∼90% (Fig. S5), as a majority of the proteoliposomes float into the 5% and 2.5% fractions of the gradient. We were therefore able to use these to study the transport mechanism of MsGabP.

**FIG 6:**
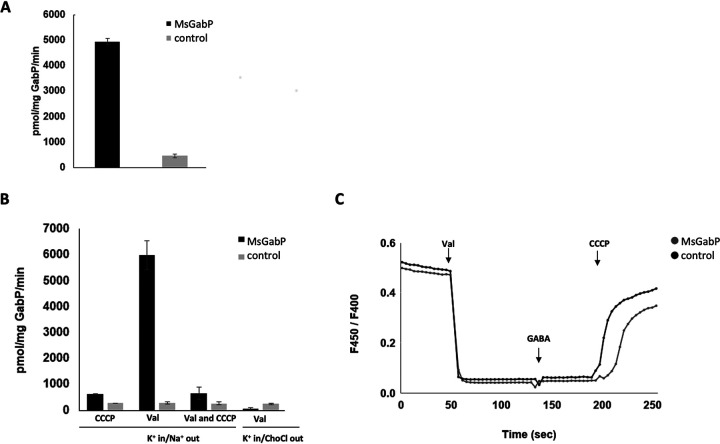
Transport activity of the purified MsGabP reconstituted into liposomes. (A and B) Radioactive GABA uptake measured in the presence of 200 mM NaCl (A) and 200 mM NaCl or 200 mM choline chloride, in the presence and absence of valinomycin (5 nM) and/or CCCP (0.03 mM) (B). GABA transport was measured for 10 min. (C) Monitoring of pH variation during GABA transport with pyranine. The ratio of fluorescence at 450/400 nm is shown over time. Bars represent uptake resulting from proteoliposomes containing MsGabP and protein-free liposome as control samples. All liposomes were loaded with 25 mM HEPES (pH 6.8), including 200 mM KCl. The data are means of three measurements. Error bars indicate standard deviations.

Radioactive transport assays showed there was a significantly higher accumulation of [^3^H]GABA in proteoliposomes containing MsGabP compared to protein-free liposomes (MsGabP versus control), when proteoliposomes loaded with K^+^ were diluted into a buffer with Na^+^ (Fig. 6A). We were unable to measure any transport of [^3^H]GABA into proteoliposomes using pH gradients alone (Fig. 6B, CCCP). In contrast, the addition of the potassium-conducting ionophore valinomycin (31) in the transport buffer (with Na^+^ present) established a negative potential inside that led to increased GABA transport compared to the uptake level in the absence of valinomycin (Fig. 6B, Val). When the membrane potential generated by valinomycin was abolished by the addition of CCCP, the uptake activity was negligible (Fig. 6B, Val and CCCP). Importantly, when choline chloride (ChoCl) was used in place of NaCl in the presence of valinomycin, GABA uptake was abolished completely, indicating that Na^+^ was essential for transport (Fig. 6B, Val, compare Na^+^ out versus ChoCl out). To confirm that the pH gradient does not contribute to GABA transport, we trapped a membrane-impermeant fluorescent pH indicator, pyranine, in the proteoliposomes. No change in pH was recorded upon the addition of GABA in MsGabP-containing proteoliposomes. This confirms that protons are not involved in GABA uptake (Fig. 6C).

### Phylogenetic analysis shows that E. coli GabP and B. subtilis GabP are closely related to MsGabP and MtbGabP.

A tblastn search against mycobacterial genomes using E. coli GabP and an E value of *e^−^*^100^ retrieved sequences across the whole span of mycobacteria, including sequences from the pathogenic species M. tuberculosis, M. ulcerans, M. avium, and M. abscessus. The primary sequences of MsGabP and MtbGabP (Rv0522) are 47% identical (56% BLOSUM62 score similar), while MsGabP and E. coli GabP are 43% identical (62% similar). We also noted that the gene product of *msmeg_5473* (Ms5473 hereafter) shares 49% identity with MtbGabP and 41% identity with MsGabP, suggesting it is also closely related to MtbGabP. Creation of a phylogenetic tree of bacterial protein sequences related to E. coli, B. subtilis, and M. tuberculosis GabP showed there are two different groups of GabP sequences, each containing members from *Proteobacteria*, *Bacilli*, and *Actinobacteria*. The first group includes the sequences from E. coli (P25527), Pseudomonas syringae (Q87UE3), Bacillus subtilis GabP (P46349), Streptomyces coelicolor (Q9L202), and Corynebacterium glutamicum AroP (Q46065), a protein characterized as an aromatic amino acid transporter. The second group has a less varied taxonomic distribution and comprises mainly *Actinobacteria* sequences, including MsGabP and MtbGabP, but also *Bacillales*, *Alphaproteobacteria*, and *Betaproteobacteria* (Fig. S6).

### Docking studies suggest that the residues involved in GABA binding are conserved between MsGabP, MtbGabP, and Ms5473.

To identify potential substrate-binding sites in the MsGabP, MtbGabP, and Ms5473 models (see Materials and Methods), molecular docking of GABA to the models with grid-based ligand docking with energetics (Glide) software (32) was performed. All predicted GABA binding poses exhibited clusters located in the same area of the protein (Fig. S7), corresponding to the outward-facing occluded conformation of the transporter as found in l-arginine/agmatine antiporter AdiC (33). The residues that interact with GABA are strongly conserved and form conserved interactions with the ligand, suggesting they are involved in substrate binding, consistent with GABA binding to all three transporters. The side chain of E119 (in MsGabP; E89 in MtbGabP) appears to hydrogen bond to the γ-amino group in GABA, while this interaction is absent in Ms5473. Instead, it appears to interact with S297, which would be a weaker interaction (Fig. S7).

## DISCUSSION

The ability of M. tuberculosis to adapt its metabolism to environmental changes, including various stress conditions, is believed to be critical for its pathogenicity (34). It is known that amino acids support growth of M. tuberculosis *in vitro* (12). However, we lack knowledge about the regulation of amino acid transport and metabolism in mycobacteria. The identity of many transporters involved in this process in M. tuberculosis is still unknown.

Here, we characterize, for the first time, the putative mycobacterial GABA permease from M. smegmatis strain MC^2^ 155. MsGabP is closely related to MtbGabP and M. bovis GabP (27) (47% sequence identity) and to the previously characterized GABA permeases from B. subtilis (35) and E. coli (24) (48% and 43% sequence identity, respectively). GABA transport is an important aspect of GABA metabolism and is regulated in concert with GABA catabolism enzymes in other bacteria. Although nitrogen-limited culture conditions induce GABA permease expression in E. coli and B. subtilis (36, 37), the regulatory mechanisms are different. As in B. subtilis (22), GabP and metabolic enzymes in mycobacteria are not physically clustered, suggesting it might exhibit functional characteristics distinct from the E. coli GabP, where there is coordinated regulation of the *gab* gene cluster (24, 38).

MsGabP was successfully expressed in C41 (DE3) Δ*acrB* cells grown in SB auto-induction medium (Fig. 1) with a yield of 0.7 mg/liter culture, extending the success of using pTTQ18-based plasmids for the expression of a range of membrane transporters (39). This provided a platform for our metabolomics and other studies. We initially used *in vivo* (using a heterologous host) targeted metabolomics to identify potential MsGabP substrates. Our metabolomics analysis suggested that GABA and serine might both be substrates of MsGabP (Fig. 2) but that asparagine, aspartate, and lysine were not substrates (see Fig. S3 in the supplemental material).

Further functional characterization using radiolabeled uptake assays with energized E. coli whole cells (Fig. 3) clearly demonstrated MsGabP-mediated GABA uptake and therefore confirmed that MsGabP is a GABA transporter. The uptake rate was in the range of ∼0.05 μmol/mg cells, but it was essentially abolished (to ∼0.01 μmol/mg cells) in the presence of 50 μM CCCP (Fig. 3). Similarly, CCCP inhibited GABA uptake by nonhomologus GabP in C. glutamicum (40). We have shown, however, that GABA uptake does not involve protons, therefore suggesting that transport is dependent on membrane potential rather than proton gradient. GABA uptake by MsGabP was also sensitive (∼70%) to competition with β-alanine (Fig. 3), as previously observed for both eukaryotic (41) and prokaryotic (42) GABA transporters. Unfortunately, we could not measure uptake of β-alanine as we could not obtain it radiolabeled. Therefore, we could not determine whether β-alanine is only a competitor or a true MsGabP substrate. Interestingly, B. subtilis GabP transports β-alanine 500 times more efficiently than the E. coli transporter, reflecting the differences in binding domains of various GABA permeases (42).

We next aimed to purify MsGabP to perform more reliable transport measurements in proteoliposomes. We screened for conditions that maintain protein stability and monodispersity prior to reconstitution. Membrane protein purification strategy success depends on the type of detergent used for solubilization and the subsequent purification steps. The on-column detergent exchange method adopted here represents a fast, versatile, and economical approach to screen a series of detergents (43–44). Solubilization trials (Fig. 4A) of membrane preparation from cells with amplified expression of MsGabP using 12 different detergents at a concentration of 1% identified DDM as the best detergent. Although DDM can extract MsGabP efficiently from the membrane, DMNPG was preferred for its ability to stabilize the protein and was therefore used in subsequent experiments.

Purified MsGabP was found to be dimeric in DMNPG (Fig. 5). This is not uncommon among this family; mouse GAT1 appears to form both dimers and high-order oligomers, as shown by *in vivo* FRET experiments (45). Li and colleagues (46) showed that Fos-choline 12 (FC-12)-purified GABA transporter from E. coli is monomeric in solution. The difference in oligomerization state is probably due to the FC-12, which is zwitterionic and much harsher than DMNPG and so presumably denatures the protein (47, 48).

We reconstituted MsGabP into proteoliposomes with 90% efficiency (Fig. S5). The presence of proteoliposomes in the upper fractions upon flotation on discontinuous sucrose gradient reflects successful protein incorporation (49). Like other secondary active transporters, substrate uptake by GABA transporter is driven by electrochemical ion gradients (50) and the cotransported ions vary among different organisms. Direct measurement of GABA transport with proteoliposomes showed that GABA uptake was tightly coupled with sodium cations (Na^+^) (Fig. 6), as previously seen in a nonhomologus C. glutamicum transporter (40).

A phylogenetic analysis (Fig. S6) of closely related sequences to GabP showed that this group of transporters is present across different classes of bacteria and includes the experimentally characterized GABA transporters of E. coli, B. subtilis, and P. syringae, although at least one member, Ncgl1062 from C. glutamicum, has been shown to be an aromatic amino acid transporter. All *Mycobacterium* species analyzed have one GabP sequence for which the phylogenetic distribution follows the evolutionary history of the genus, suggesting that it was present in the last common ancestor of all mycobacteria. It is reasonable to assume that in this orthologous cluster, the ability to transport GABA is preserved and that the annotation of Rv0522 as a GABA permease is appropriate. Some mycobacteria have a second GabP sequence that was probably acquired by lateral gene transfer from within the same group of transporters; this includes M. smegmatis. Here, we have shown that this second member is also a GABA transporter. Finally, structural analysis of the docked complexes revealed a similar binding pocket for GABA in all three homologues, with GABA being recognized by conserved amino acids residues (Fig. S7). The poses for MsGabP and MtbGabP are more similar than that of Ms5473, supporting our argument that both MsGabP and MtbGabP are GABA transporters. We can only speculate, but MsGabP and Ms5473 may differ in when they are expressed and/or in their relative affinities for GABA, as is the case in Staphylococcus aureus, which has two proline transporters (51).

GABA may be an important metabolite required for mycobacterial pathogenesis, raising the therapeutic potential of inhibitors toward GABA permease. We hope these studies will lead to structural and further biochemical exploration of this novel mycobacterial transporter, resulting in the discovery of new potent drugs against TB.

## MATERIALS AND METHODS

### General.

The primers used for PCR were from Sigma-Aldrich (St. Louis, MO); enzymes for cloning were from New England BioLabs (Ipswich, MA); the reagents for the bicinchoninic acid (BCA) assay were from Thermo Fisher Scientific (Waltham, MA); His tag-horseradish peroxidase (HRP)-conjugated antibody was from Bio-Techne (Minneapolis, MN); detergents were from Anatrace (Maumee, OH); and radiolabeled γ-[2,3-3H(N)]-aminobutyric acid ([^3^H]GABA) was from Perkin Elmer (Waltham, MA). All other chemicals were from Sigma-Aldrich (St. Louis, MO) and were of analytical grade or better. The Mycobacterium smegmatis MC^2^ 155 strain was purchased from ATCC (Manassas, VA), C41 (DE3) Δ*acrB* was from Lucigen Corporation (Middleton, WI), BL21 Gold (DE3) was from Stratagene (La Jolla, CA), BL21 Star (DE3) was from Thermo Fisher Scientific (Waltham, MA), and plasmid pRARE2 was from Novagen (the Merck Group, Darmstadt, Germany). All media, buffers, and other solutions were prepared using deionized water. All media were sterilized by autoclaving or, for thermally sensitive solutions, by passage through 0.2-μm filters from Millipore.

### Cloning.

An expression vector pL33 for the production of MsGabP was constructed using the vector pTTQ18 (52). pL33 encodes a C-terminal His tag preceded by an HRV-3C protease cleavage site (53). The gene *msmeg_6196* encoding GabP was amplified with M. smegmatis MC^2^ 155 genomic DNA as the template by PCR using the upstream primer with NheI site, 5′-GCTAGCCTCGAATCGAGATCCGATCTG-3′, and downstream primer with *Sbf*I site 5′-CCTGCAGGCTCATCGGTTCTCGCAGC-3′. The amplified fragment was digested with NheI and *Sbf*I and inserted between the corresponding sites of plasmid pL33 to construct the expression plasmid pL33-MsGabP.

### Cell growth and membrane preparation.

Expression tests for MsGabP were performed using E. coli strains C41 (DE3) Δ*acrB*, C41 (DE3) Δ*acrB* pRARE2, C43 (DE3) Δ*acrB* pRARE2, BL21 Gold (DE3) pRARE2, and BL21 Star (DE3) pRARE2 and three different types of medium for cell growth (lysogeny broth [LB], superbroth [SB], and M9 autoinduction medium) in 24-deep-well plates (Whatman plc, GE Healthcare, IL) at 37°C for 24 h with shaking at 1,300 rpm (54). Isopropyl-β-d-1-thiogalactopyranoside (IPTG) induction and autoinduction were compared to determine which condition produced more of the target protein. Lysed cells (10 μg) (54) were loaded onto SDS-PAGE gels and protein expression was determined by Western blotting using His tag-horseradish peroxidase-conjugated antibody. Large-scale expression of MsGabP was performed in a 30-liter fermentor (Infors HT). The cells were grown in SB autoinduction medium at 37°C for 24 h and harvested by centrifugation (6,000 × *g*, 20 min, 4°C). The cells were resuspended in 1× phosphate-buffered saline (PBS) buffer (10 mM Na_2_HPO_4_, 1.8 mM KH_2_PO_4_, 137 mM NaCl, and 4 mM KCl [pH 7.4]) with a ratio of 6 ml buffer/g cells. Membranes were prepared following the protocol in reference 54 and resuspended with 1× PBS buffer and total protein concentration in the membrane was measured by BCA assay (55).

### Solubilization and purification of MsGabP.

The solubilization test was carried out at 4°C with 12 different detergents (Table 1). The membrane fraction of E. coli/MsGabP adjusted to 2 mg/ml was incubated in 20 mM Tris (pH 8.0), 150 mM NaCl, 10% (vol/vol) glycerol, and 1% (wt/vol) of the tested detergent at 4°C for 1 h. Samples before and after centrifugation at 100,000 × *g* for 1 h were analyzed by Western blotting. Purification was started with 100 mg of total membrane protein that was homogenized and solubilized for 1 h at 4°C in solubilization buffer (10 mM Na_2_HPO_4_, 1.8 mM KH_2_PO_4_, 4 mM KCl, 287 mM NaCl, 7.5 mM imidazole, 10% [vol/vol] glycerol, pH 7.4) with 1% (wt/vol) DDM at a protein concentration of 5 mg/ml, followed by removal of insoluble material by centrifugation at 100,000 × *g* for 1 h. The supernatant was incubated with 1 ml HisPur cobalt resin (50% slurry) (Thermo Fisher Scientific, Waltham, MA) and then preequilibrated with wash buffer 1 (same composition as the solubilization buffer) containing 0.05% (wt/vol) DDM at 4°C for 2 h with gentle mixing. Immobilized-metal affinity chromatography (IMAC) was performed by mixing the supernatant with the equilibrated resin for 2 h with gentle mixing. The resin was then packed into the Econo-Pac disposable gravity-flow chromatography column (Bio-Rad, Hercules, CA). Unbound material was collected followed by washing of the column with ∼10× column volumes of wash buffer 1 and 2 (differing from wash buffer 1 only by the imidazole concentration, which was 10 mM).

### On-column detergent exchange.

Detergent was exchanged on column from DDM into CYMAL 6, β-OG, OGNPG, DMNPG, LMNPG, DM, UDM, UDTM, DDTM, 13M, and 14M by replacing DDM in the wash buffer 2 with 3 × CMC of each detergent. The resin then was washed with a wash buffer 3 (20 mM HEPES [pH 7.0], 100 mM NaCl, 5% (vol/vol) glycerol, 3 × CMC of detergent) for 8× column volume to remove imidazole. HRV-3C protease was then added at a molar ratio of 1:1 of the target protein to the resin with a minimal volume of wash buffer 3 and incubated at 4°C overnight to cleave the His tag. The following day, the protein was eluted from the column using ∼7 ml of wash buffer 3 and then concentrated to a volume of ∼100 μl by centrifugation using a concentrator with a molecular weight cutoff of 50 kDa (Vivaspin 2; Sartorius).

### Microscale fluorescent thermal stability assay.

The stability of the protein purified in different detergents was checked by microscale fluorescent thermal stability assay as described previously (28), with the following modifications. The buffer used for dilution of the dye *N*-[4-(7-diethylamino-4-methyl-3-coumarinyl)phenyl] maleimide (CPM) was the same as the buffer that the protein sample was eluted with. After briefly mixing the dye and the protein sample, the mixture was equilibrated at room temperature for 10 min while protecting it from light and then placed into the Stratagene Mx3005P Real Time PCR machine (Agilent Technologies, Santa Clara, CA). The ramp rate was 4°C/min and the starting and ending temperatures were 25°C and 90°C. Data were processed with GraphPadPrism for Mac (GraphPad Software, San Diego, CA [http://www.graphpad.com/scientific-software/prism/]).

### Size exclusion chromatography coupled with multiangle laser light scattering.

The molecular mass and the oligomerization state of the purified MsGabP was determined via size exclusion chromatography coupled to light scattering, absorbance, and differential refractive index detectors method. The refractive index and light scattering detectors were from Wyatt Technology (Goleta, CA) and the UV detector and chromatography pumps from Shimadzu Corporation (Kyoto, Japan). The Superose 6 column (WTC-MP030S5; Wyatt Technology, Goleta, CA) was equilibrated with 20 mM HEPES (pH 7.0), 100 mM NaCl, 5% (vol/vol) glycerol, 0.0102% (wt/vol) DMNPG overnight. Thirty microliters of MsGabP (3 mg/ml) was injected and the sample eluted from the column was analyzed by three detectors (30). Data obtained were analyzed using ASTRA software package, version 6.1 (Wyatt Technology, Goleta, CA). The program calculated M_W,protein_, M_W,detergent_, and M_W,total_ throughout the peak and also provided information on the monodispersity of the peak (56).

### Targeted metabolomics study.

E. coli strain C41 (DE3) Δ*acrB* harboring pL33 or pL33-MsGabP was cultured in 50 ml of LB medium at 37°C for 8 h in 250-ml shaker flasks. Cells (10^8^) were transferred onto mixed cellulose filters (pore size 0.22 μm; Merck Millipore, Billerica, MA) by vacuum filtration and then incubated overnight at 30°C in agar plates (1.5% (wt/vol) containing minimal medium (50 mM Na_2_HPO_4_, 50 mM KH_2_PO_4_, 25 mM [NH4]_2_SO_4_, 2 mM MgSO_4_, trace metals, vitamins, and 0.5% glucose) supplemented with ampicillin (100 μg ml^− 1^). The cells were adapted for 1 h at 37°C. Filters with the cells were transferred onto minimal medium agar (1.5% [wt/vol]) plates supplemented with the specific amino acid tested (1 mM or 5 mM), 0.5 mM IPTG, and ampicillin (100 μg ml^− 1^), and incubated for 0.5 h at 37°C. The cells were harvested and transferred to 1 ml acetonitrile-methanol-water (2:2:1 vol/vol/vol) solution. The cells were then disrupted using a bead beater and polar metabolites were extracted. After centrifugation, supernatants were collected and filtered with 0.22-μm spin-X column filters (Costar, Corning Inc., NY). Extracts were stored at −80°C before analysis. The amino acids tested were arginine, aspartate, asparagine, GABA, glutamate, glutamine, lysine, and serine. The liquid chromatography–mass spectrometry was performed as described previously (57). Aqueous normal phase liquid chromatography was performed using an Agilent 1200LC system with a flow rate of 0.4 ml/min. Elution of polar compounds was performed using a gradient of solvents A (Milli-Q water and 0.2% acetic acid) and B (acetonitrile and 0.2% acetic acid). Accurate mass spectrometry was performed using an Agilent Accurate Mass 6230 TOF apparatus equipped with a multimode ion source. Data were analyzed using Qualitative Analysis B.07.00 software and the metabolites were identified comparing the accurate *m/z* (error less than 10 ppm) and the retention time with the accurate *m/z* and the retention time of standard solutions for the specific metabolite. The ions counts were recorded and normalized to the residual protein content (detected by BCA assay) present in each extract.

### Whole-cell radiolabeled assay.

C41 (DE3) Δ*acrB* E. coli was grown in M9 minimal medium supplemented with glycerol (20 mM) and carbenicillin (100 μg ml^− 1^) in volumes of 50 ml at 37°C in 250-ml baffled conical flasks with aeration at 200 rpm to an optical density at 680 nm (OD_680_) of ∼0.4 to 0.6. The cells were then either uninduced or induced with IPTG (0.5 mM) for a further 1 h. Harvested cells (by centrifugation at 2,500 × *g* for 10 min) were washed three times with 40 ml of buffer (5 mM morpholineethanesulfonic acid [MES; pH 6.6], 100 mM NaCl, and 50 mM KCl) and then resuspended to a cell density of *A*_680_ of ∼2.0. The cell suspension (196 μl) containing 20 mM glycerol was aerated in a bijou bottle held in a water jacket at 37°C for 3 min, and then [^3^H]GABA (50 μM) with a specific activity of 10 μCi/ml was added with brief mixing. Exactly 10 min after adding the [^3^H]GABA, 80-μl aliquots were transferred to cellulose nitrate filters (0.45 μm pore size), presoaked in transport buffer on a vacuum manifold and washed with transport buffer (6 ml). The filters were transferred to scintillation vials with 10 ml emulsifier-safe liquid scintillation fluid (Perkin Elmer) and incubated overnight. The level of ^3^H radioactivity was measured by liquid scintillation counting (Packard Tri-Carb 2100TR instrument; Perkin Elmer, Waltham, MA). The measured value of disintegrations per minute was converted into micromoles per milligram of cells. Background counts were measured by washing filters under vacuum in the absence of cells or radiolabeled substrate. Standard counts were measured by transferring 1, 2.5, 5, and 10 μl of radiolabeled substrate stock solution directly to a washed filter in the vial.

To test the effect of CCCP on transport, it was added at a final concentration of 50 μM. To test the effect on [^3^H]GABA uptake, unlabeled β-alanine (final concentration, 5 mM) was added to the cells prior to energizing the proteoliposomes. [^3^H]GABA was added 5 min after and an 80-μl aliquot was taken to measure the radioactivity.

### Protein reconstitution and liposome flotation assay.

The E. coli lipid extract (Avanti Polar Lipids, Inc.) dissolved in chloroform was dried under nitrogen and the lipid film was resuspended by vortexing to 10 mg/ml with reconstitution buffer (25 mM HEPES [pH 6.8], including 200 mM KCl and 1 mM dithiothreitol [DTT]). The dissolved lipid was then passed 11 times through 0.4-μm- and then 0.2-μm-pore-size filters (polycarbonate Nuclepore Track Etch membrane filters) placed inside the barrel of the extruder. To destabilize the liposomes and allow the insertion of the membrane protein, 1.1% β-octyl glucoside (OG) was added to 1 ml of liposomes. The purified protein was added at 500:1 lipid to protein ratio and incubated at 4°C for 1 h. This was then diluted to 15 ml with a reconstitution buffer and centrifuged at 100,000 × *g* for 1 h at 4°C. Liposome pellets were resuspended in 1 ml of the reconstitution buffer. Proteoliposomes were analyzed using a flotation assay in a sucrose gradient made of layers containing 60%, 30%, 10%, 5%, and 2.5% (mass/vol) sucrose. Proteoliposomes (250 μl) were added to 250 μl of 60% sucrose (in 1× HEPES buffer, pH 6.8). This fraction was overlaid with 0.5 ml of 30% sucrose, 20%, 10%, 5%, and finally 0.4 ml of 2.5% sucrose. After centrifugation at 259,000 × *g* for 16 h, the fractions were collected from the gradient, and analyzed for protein by SDS-PAGE.

### Fluorimetric transport assay.

The proteoliposomes (5 μl) loaded with 25 mM HEPES (pH 6.8), 200 mM KCl, and 1 mM pyranine dye were diluted in a buffer (1 ml) containing 25 mM HEPES (pH 6.8) and 200 mM NaCl and equilibrated in a stirred cuvette in the Photon Technology International QM-1 spectrophotometer (PTI, United Kingdom) for 3 min. Fluorescence was monitored using 400 and 450 nm excitation and 509 nm emission. To induce membrane potential, valinomycin was added (5 nM) at ∼60 s, followed by the addition of 30 mM GABA at ∼160 s and 0.03 mM CCCP at ∼200 s. The internal pH change was monitored as a change in the ratio of 450/400 nm pyranine fluorescence (58).

### Radiolabeled assay with proteoliposomes.

GABA transport was initiated by diluting 6.6 μl of proteoliposomes loaded with 25 mM HEPES (pH 6.8)–200 mM KCl buffer into 330 μl of external transport buffer: either 25 mM HEPES (pH 6.8)–200 mM NaCl buffer or 25 mM HEPES (pH 6.8)–200 mM choline chloride. To determine whether the presence of membrane potential (ΔΨ) was needed to drive GABA uptake, 5 nM valinomycin and/or 0.03 mM CCCP were added to the transport buffers. The incubation time was about 60 s before starting the reactions by the addition of [^3^H]GABA (specific activity, 10 μCi/ml) at a final concentration of 50 μM. At 10 min after adding the radiolabeled substrate, 80-μl aliquots were transferred to cellulose nitrate filters (0.45 μm pore size) presoaked in transport buffer on a vacuum manifold and washed immediately with transport buffer (6 ml). The filters were transferred to scintillation vials and radioactivity was measured as described for the whole-cell assay. The measured value of disintegrations per minute was converted into picomoles per milligram of protein per minute.

### Phylogenetic analysis.

The sequences of Cluster of Orthologous Groups 1113 were downloaded from EggNOG (59) and clustered using an 80% identity threshold with CD-HIT (60). An alignment was calculated using Mafft v7.310 FFT-NS-2 (61). Sequences with less than 400 residues were removed and the alignment was simplified to less than 85% redundancy with Jalview 2.11 (62). Both termini were truncated to the first and the last column with 100% occupancy.

Independently, in-house scripts and BLAST+ (63) were used to mine mycobacterial and selected model bacteria sequences using the sequence of E. coli GABA permease as query and an expected threshold of *e*^−100^. Matches were aligned with MUSCLE (64) and the alignment was truncated and simplified to less than 95% redundancy as previously explained. Both alignments were combined using Mafft and checked for redundancy and partial/truncated and very divergent entries were removed.

The cluster of GabP sequences was then extracted, leaving behind those of related the transporters, e.g., AnsP (P9WQM7), CycA (O33203), YifK (P27837), ProY (P0AAE2), pheP (P24207), LysP (P25737) and YvbW (O32257). A final alignment of this cluster was calculated using Mafft G-INS-I and consisted of 273 sequences. The best-fit evolutionary model for the alignment was LG+F+R6 as calculated by ModelFinder (65). Maximum likelihood phylogenetic analysis was done using IQ-TREE v1.6.11 (66) with 100 standard bootstrap replicates. Phylogenetic trees were visualized with Dendroscope (67).

### Molecular model of GabPs and molecular docking of GABA.

*De novo* protein modeling of MsGabP was performed using the Robetta server (68). The protein structures of MtbGabP and Ms5473 were homology-modeled by Phyre2 (69) using the MsGabP protein sequence as query. The rank matches for both models had 50% identity and 100% confidence, indicating high probability of modeling success. The graphical user interface Maestro (version 12.4; Schrodinger LLC, New York, NY) was used to visualize the three protein models, which were prepared for molecular modeling using the Protein Preparation Wizard (default settings). The GABA ligand was drawn in Maestro and a low-energy, zwitterionic conformation was generated using Ligprep (version 12.4; Schrodinger LLC, New York, NY). A 36 Å receptor grid encompassing the protein structure was generated, centered on a user-defined residue (MsGabP G223, MtbGabP T193, and Ms5473 V210). Molecular docking of the GABA ligand was performed on each protein model using Glide in SP mode with default settings but outputting 15 poses (version 12.4; Schrodinger LLC, New York, NY). The predicted binding poses of the GABA ligand were ranked by docking score and the list was visually inspected for predicted hydrogen bonding and the associated directionality and length, steric clashes/unfavorable interactions, and ligand conformations. A GABA-binding pose within each protein model was chosen based on the above criteria.

## Supplementary Material

Supplemental file 1
