# Supplementary material for: Functional Characterization of the γ-Aminobutyric Acid Transporter from Mycobacterium smegmatis MC2 155 Reveals Sodium-Driven GABA Transport
Source: J Bacteriol. 2021 Jan 25;203(4):e00642-20. doi: 10.1128/JB.00642-20 (PMC7847548; doi:10.1128/JB.00642-20)
Supplement: Supplemental file 1 [file JB.00642-20-s0001.pdf]

SUPPLEMENTAL MATERIAL

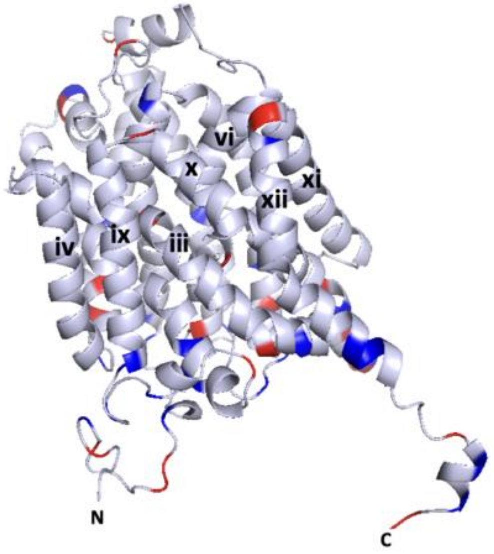

FIG S1 *De novo* three-dimension model of MsGabP built with the Robetta server. Positively-charged amino acids are coloured blue and negatively charged ones, red.

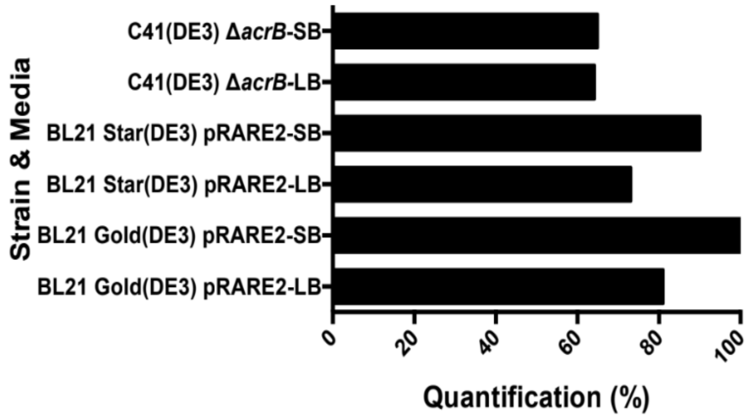

FIG S2 Western blot quantification (performed by ImageJ) of screening expression results with strains C41 (DE3) Δ*acrB*, BL21 Star (DE3) pRARE2 and BL21 Gold (DE3) pRARE2. The values were calculated with numbers obtained from ImageJ divided by the number from the condition: BL21 Gold (DE3) pRARE2 and SB auto-induction media (the highest expression level).

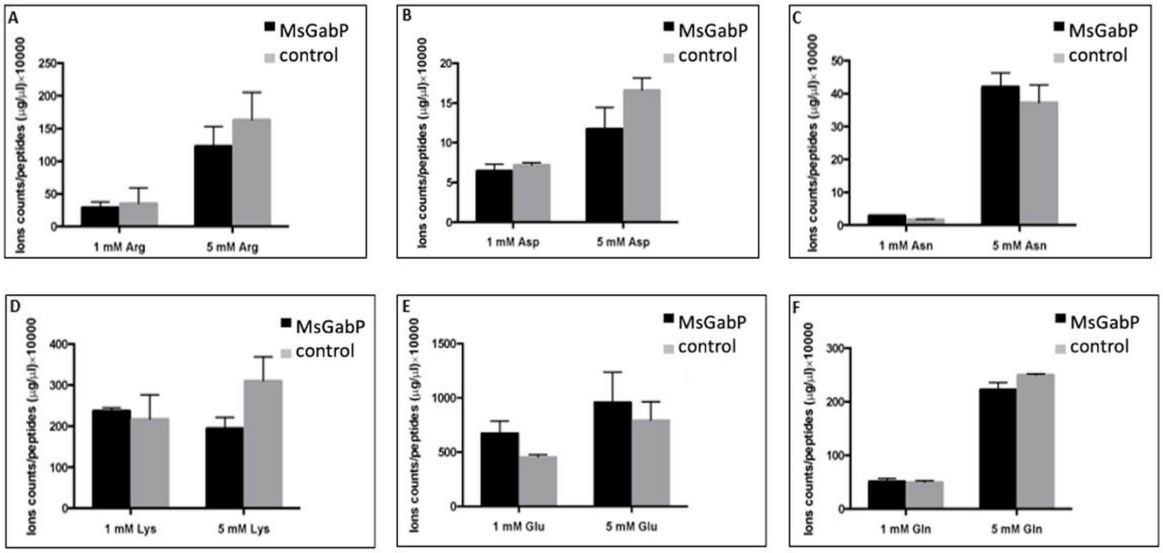

FIG S3 Targeted metabolomics study results of (A) arginine, (B) aspartate, (C) asparagine, (D) lysine, (E) glutamate and (F) glutamine. The amino acids were tested with two concentrations, 1 mM and 5 mM. The black and grey bar represents uptake resulted from cells harbouring plasmid MsGabP or empty vector as control, respectively. The charts show means (bars) and standard deviations (error bars) of three biological replicates of one independent experiment. They are representative of two independent experiments. The y-axes report the ions counts normalised on residual protein content (ions counts/peptides*10000).

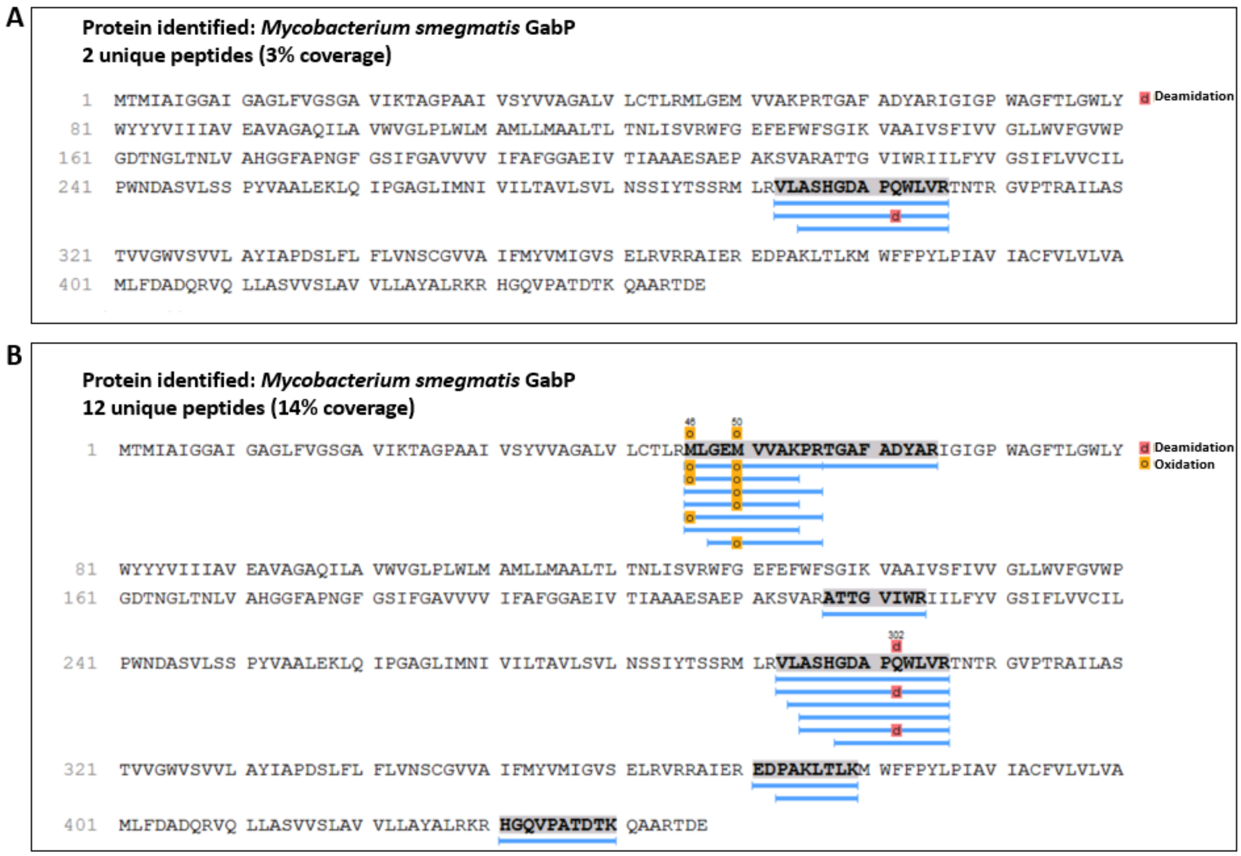

FIG S4 Protein sequence coverage by mass spectrometry of the MsGabP excised from two SDS-PAGE gel bands (bands are shown in Fig. 5A). Trypsin-generated fragments resulted in identification of 3% of MsGabP from the upper band (A) and 14% from the lower band (B). Blue lines correspond to digested peptides identified in MsGabP sequence. There were twelve and two high confidence supporting peptides identified to be mapped to only one protein group. Amino acids that have post translation modification sites are identified and marked with a letter *o* in a yellow square (oxidation sites) and with a letter *d* in a pink square (deamidation sites).

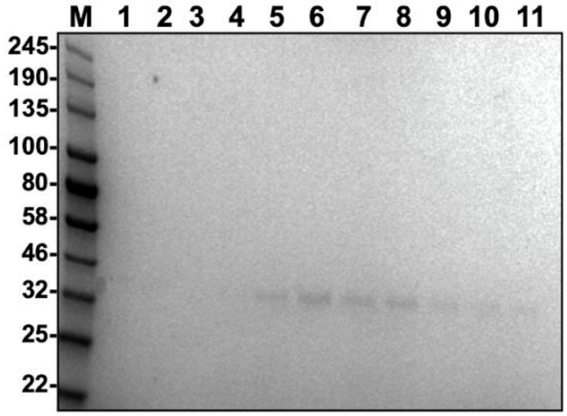

FIG S5 SDS-PAGE of the discontinuous sucrose flotation assay fractions demonstrating reconstitution of MsGabP. Fractions were loaded as follows: M: protein molecular marker (kDa); 1: 60%; 2-4: 30%; 5: 10%; 6-8: 5%; 9-11: 2.5%.

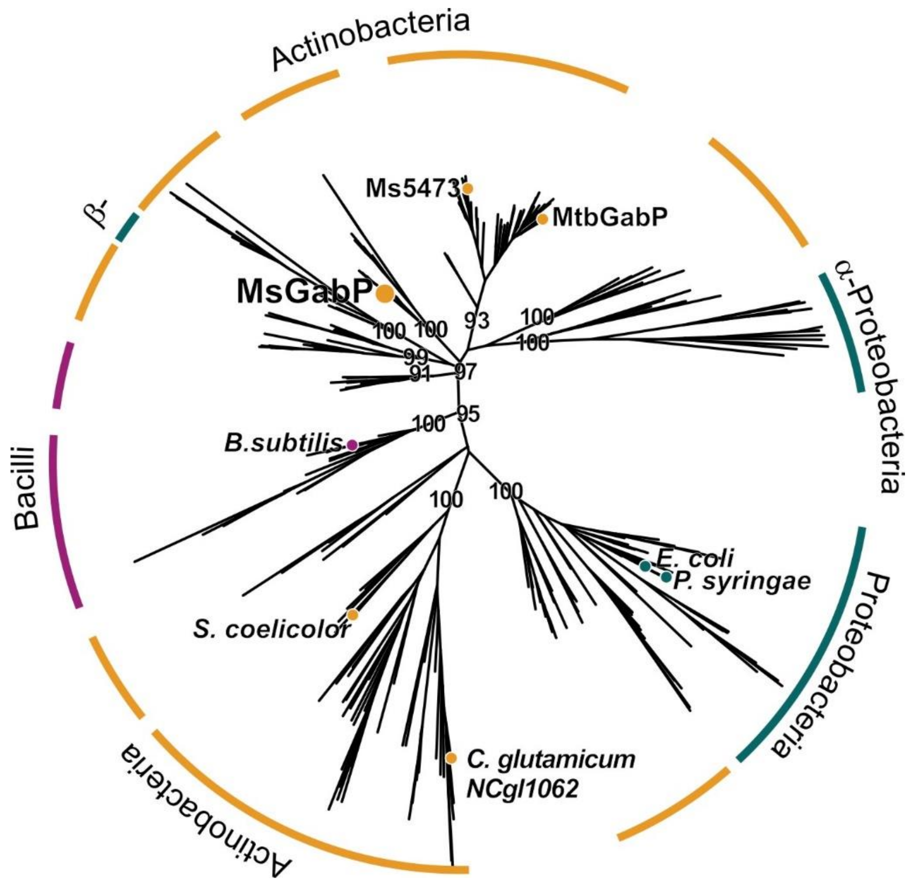

FIG S6 Maximum-likelihood phylogenetic tree of bacterial putative and characterized GABA transporters and closely related sequences. Sequences belonging to species in the class Bacilli are labelled in magenta, those in the Phylum Proteobacteria are in cyan and those in the Phylum Actinobacteria are in gold. MsGabP is the GABA transporter here characterized. MtbGabP is the putative GABA transporter in *M. tuberculosis* and Ms5473 is the orthologue of MtbGabP.

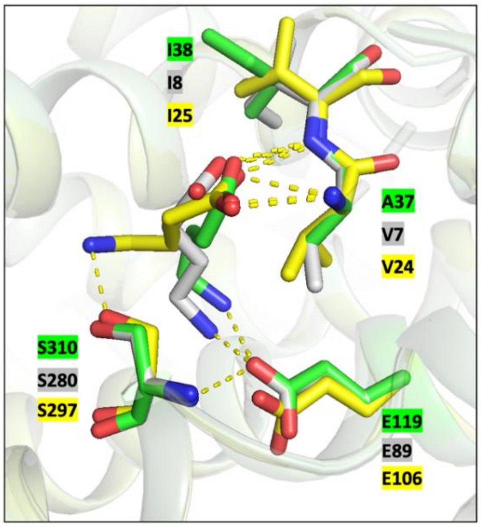

FIG S7 Superposition of the Molecular Docking of GABA binding to the three transporters: MsGabP (green), MtbGabP (grey), Ms5473 (yellow) showing that MtbGabP and MsGabP appears to be more similar to each other than they are to Ms5473. All three of the structures were predicted. Key residues involved in binding of GABA are shown in stick model and labelled. The hydrogen bonds are indicated by yellow dashes. Atom colors: O in red, N in blue, and C in protein backbone color.
